# Establishing Infodemic Management in Germany: A Framework for Social Listening and Integrated Analysis to Report Infodemic Insights at the National Public Health Institute

**DOI:** 10.2196/43646

**Published:** 2023-06-01

**Authors:** T Sonia Boender, Paula Helene Schneider, Claudia Houareau, Silvan Wehrli, Tina D Purnat, Atsuyoshi Ishizumi, Elisabeth Wilhelm, Christopher Voegeli, Lothar H Wieler, Christina Leuker

**Affiliations:** 1 Risk Communication Unit Robert Koch Institute Berlin Germany; 2 Department for Infectious Disease Epidemiology Robert Koch Institute Berlin Germany; 3 Centre for Artificial Intelligence in Public Health Research Robert Koch Institute Berlin Germany; 4 Health Emergencies Programme Department of Pandemic and Epidemic Preparedness and Prevention World Health Organization Geneva Switzerland; 5 School of Public Health Information Futures Lab Brown University Providence, RI United States; 6 Centers for Disease Control and Prevention Atlanta Georgia; 7 Robert Koch Institute Berlin Germany; 8 Digital Global Public Health Hasso Plattner Institute University of Potsdam Potsdam Germany

**Keywords:** infodemic, social listening, communication, infodemiology, public health, health promotion, misinformation, integrated analysis, infodemic insights

## Abstract

**Background:**

To respond to the need to establish infodemic management functions at the national public health institute in Germany (Robert Koch Institute, RKI), we explored and assessed available data sources, developed a social listening and integrated analysis framework, and defined when infodemic management functions should be activated during emergencies.

**Objective:**

We aimed to establish a framework for social listening and integrated analysis for public health in the German context using international examples and technical guidance documents for infodemic management.

**Methods:**

This study completed the following objectives: identified (potentially) available data sources for social listening and integrated analysis; assessed these data sources for their suitability and usefulness for integrated analysis in addition to an assessment of their risk using the RKI’s standardized data protection requirements; developed a framework and workflow to combine social listening and integrated analysis to report back actionable infodemic insights for public health communications by the RKI and stakeholders; and defined criteria for activating integrated analysis structures in the context of a specific health event or health emergency.

**Results:**

We included and classified 38% (16/42) of the identified and assessed data sources for social listening and integrated analysis at the RKI into 3 categories: social media and web-based listening data, RKI-specific data, and infodemic insights. Most data sources can be analyzed weekly to detect current trends and narratives and to inform a timely response by reporting insights that include a risk assessment and scalar judgments of different narratives and themes.

**Conclusions:**

This study identified, assessed, and prioritized a wide range of data sources for social listening and integrated analysis to report actionable infodemic insights, ensuring a valuable first step in establishing and operationalizing infodemic management at the RKI. This case study also serves as a roadmap for others. Ultimately, once operational, these activities will inform better and targeted public health communication at the RKI and beyond.

## Introduction

### The Infodemic

Over the last few decades, our information ecosystem has undergone changes and shifts, where the general public moved away from traditional media and institutions as a primary source of health information to a more decentralized model with many different sources of information [1]. Different groups and generations have their own networks, information sources, and ways of interacting and sharing information in a digitally connected, increasingly polarized world [2]. The COVID-19 pandemic has made these trends increasingly clear and has been accompanied by an *infodemic*—too much information, including false or misleading information in digital and physical environments during a health emergency [3]. The lack of agreement between different information sources, as well as different levels of trust in different sources by different people, can cause uncertainty in the general population and impact the effectiveness of risk communication. There is more room for misinformation and disinformation to spread, for trust in public policy and political actions to be undermined [3], and for public health measures to be jeopardized [4]. Initial studies evaluating the COVID-19 pandemic response have acknowledged the need for greater investment in risk communication and community engagement strategies to foster trust in public health guidance and ultimately improve adherence to public health guidance and health decision-making [5-8]. Consistent with these findings, trust in institutions has been strongly linked to responsiveness and reliability in delivering policies and services [9]. Therefore, one of the key recommendations of the Organisation for Economic Co-operation and Development (OECD) is to connect and engage better with citizens in policy design, delivery, and reform, and ensure the inclusion of people at a higher risk of negative health impacts from infodemics. Infodemic management aims to achieve this through social listening and community engagement, as well as targeted public health messaging.

### Infodemic Management

One way to support people in making informed health decisions is to provide responsive, evidence-based, and target-group-specific risk and health communication [10]. These communications must correspond to people’s concerns and questions. Moreover, people need to be equipped with the right tools to find reliable information, identify misinformation [11], and assess the quality of (scientific) evidence. Well-planned and executed infodemic management can help develop the right messages for the right target groups at the right time as well as boost people’s health and scientific literacy [12]. Although the terms *infodemiology*, *infodemic*, and *infoveillance* have existed for a long time [13,14], the field of infodemic management and its line of research have now been formally acknowledged by public health organizations as a novel, emerging scientific field and a critical area of practice during a pandemic [15,16]. Responding to *narratives about* the virus requires an approach similar to responding to the spread of the virus itself, including (early) detection, diagnosis, and identification of appropriate responses and interventions [17]. As both should occur early and in parallel, the European Centre for Disease Control and Prevention (ECDC) updated its guidance document in 2022 by adding infodemiology and infodemic management to the core competencies in applied infectious disease epidemiology [18]. Moreover, in 2023, the World Health Organization (WHO) is convening panels to develop WHO guidance and ethical considerations for social listening and integrated analysis, as well as WHO guidance on social listening and integrated analysis for public health, with an application to acute respiratory disease.

### Social Listening and Integrated Analysis

Risk communication and community engagement are crucial elements of pandemic response [7]. Effective communication starts with listening; therefore, social listening is an essential tool in the infodemic management toolkit. Social listening is defined as monitoring the understanding, questions, concerns, information voids, narratives, misinformation, and disinformation that circulate in both web-based and offline environments (Textbox 1) [19,20]. Although it is a common practice for businesses to engage in digital marketing and monitor social media channels for mentions of their brand, competitors, or products [21], social media monitoring is just starting to find its way into the public sector. The increase in web-based communications has, in combination with computational power and artificial intelligence (AI), enabled real-time social listening, as implemented with the pilot Early AI-supported Response with Social Listening platform that tracks web-based COVID-19 conversations [22,23]. In addition to monitoring web-based conversations, offline social listening (including traditional media and other sources such as user search trends, epidemiological data, and socio-behavioral data) can be used to understand ongoing narratives at the population level [24].

Integrated analysis extends web-based social listening by considering data sources beyond social media (Textbox 1). These include news articles, Google searches, primary research, community dipstick surveys, citizen questions posed via hotlines, monitoring or surveillance reports, epidemiological and behavioral data, surveys and polls, and many more. Any data source that can provide insight into behaviors, questions, concerns, information voids, circulating narratives, misinformation, and disinformation (Textbox 1) within a given population for a given public health event was eligible. In the integrated analysis, different data sources were combined to identify themes and narratives across data sources. One advantage of integrated analysis is that it is less biased toward social media users and includes more diverse population groups. Another advantage is that a specific theme’s importance may be judged more easily through triangulation (eg, if the same theme comes across many different sources). The scope of an integrated analysis can be varied based on current challenges and goals and available resources, for example, one could monitor and assess narratives around COVID-19 and monkeypox (mpox) as a whole (WHO Infodemic Insights reports for COVID-19 [23,24] and mpox [25]) or focus on vaccines and vaccine confidence (US Centers for Disease Control and Prevention [US CDC] State of Vaccine Confidence Insights Reports for COVID-19 [26] and mpox [27]).

Identifying and understanding the information voids, narratives, and sentiments behind conversations regarding public health issues through social listening and integrated analysis can help design adapted and targeted risk communication messages. These risk communication messages can have several aims: to prevent the circulation of misinformation by prebunking anticipated misinformation narratives, or responding to them if necessary, counteract stigma against affected groups [28,29], fill information voids, promote resilience, or contribute to behavioral change. Social listening and integrated analysis also have the power to identify research gaps and programmatic bottlenecks that the public perceives as a challenge (including access barriers), as well as guidance that confuses people or could potentially erode trust. These infodemic insights can point out confusion where the health authority is experiencing communication failures with the public, and what policy or programmatic levers can be used to address it beyond risk communication activities.

**Infodemic management terminology used in this work.**

- Infodemic: an overabundance of information—some accurate and some not—that occurs during an epidemic [3,15].
- Infodemic management: the systematic use of risk- and evidence-based analysis and approaches to manage the infodemic and reduce its impact on health behaviors during health emergencies. Infodemic management aims to enable good health practices through four types of activities: (1) listening to community concerns and questions; (2) promoting understanding of risk and health expert advice; (3) building resilience to misinformation; and (4) engaging and empowering communities to take positive action [30].
- Infodemiology: the epidemiology of information; describing and analyzing information and communication patterns and their relationship to population health status [13].
- Infodemic insights: findings or conclusions from a data source (report) that has its own analysis plan that is tailored to the data type, source, and context of where the data are collected and the population it covers [24]. It is used to make recommendations for action, for more effective engagement.
- Infoveillance: using infodemiology data for surveillance [13,14].
- Social listening or infodemic surveillance [17], sometimes used as a synonym for “infoveillance”: Monitoring different web-based data (e social media) and offline data (traditional media and other sources such as user search trends, epidemiological data, and socio-behavioral data) sources to understand population understanding, perceptions, concerns and questions, information voids, narratives, misinformation and disinformation, and other relevant information about people’s reactions to a health topic [24].
- Integrated analysis: a planned methodological examination of different types of data sources that combine social listening intelligence with other types of information (eg, health seeking behavior, health service use, epidemiology, fact-checking and information seeking trends, and mobility reports) to produce infodemic insights [24].
- (Infodemic) insights report: a reporting output of integrated analysis that contextualizes findings from social listening and other data sources for use by health authorities to act based on a planned methodological frame for prioritization of actions and interventions. Important elements to include are a diagnosis of barriers and facilitators to desired behavior and how possible recommended actions support desired public health behaviors, which may be internal to the health system and externally facing strategies [24].
- Misinformation: false information, regardless of the intent to mislead [11].
- Disinformation: misinformation that is deliberately disseminated to mislead [11].

### The German Context

In Germany, the Federal Centre for Health Education (*Bundeszentrale für gesundheitliche Aufklärung* [*BZgA*]) [27] is tasked with health education and promotion focused on the public. During the COVID-19 pandemic, an increasing number of citizens have also turned to the national public health institute—the Robert Koch Institute (RKI)—as well as to communications by the Federal Ministry of Health for behavioral advice and information on the pandemic. One indicator of this is the number of daily visitors on the RKI website, which has increased from ~30,000 in early February 2020 to an average of ~250,000 to 350,000 visits per day since the end of May 2020. The number of visits peaked on March 16, 2020, with 1,685,000 visits. The RKI’s follower count on Twitter (@rki_de) has increased from 12,000 (January 1, 2020) to 600,000 as of the time of writing (October 2022).

At the end of 2021, the German chancellor convened a scientific expert council of 19 members from different disciplines to develop evidence-based proposals to help curb the spread of the virus and tackle the pandemic [31]. In their fifth statement (January 20, 2022), the council unanimously called for the implementation of coordinated risk and health communication practices [4], which are also consistent with key infodemic management principles: (1) *generating* the best available knowledge to date (eg, through monitoring media and the extent to which the public takes up health-relevant behaviors), (2) *translating* relevant data, statistics, and indicators into behaviorally relevant advice for different target groups (Who is reached via which medium and format? How does information complexity need to be adapted?) and countering misinformation and disinformation; (3) *disseminating* communications via multiple channels, making use of web-based and offline media, influencers (by providing them with adequate materials), eHealth offers (such as web-based consultations) and collaborating with science communicators; and (4) *evaluating* the aforementioned measures and using the results for continuous quality improvement. As the council says, “in a decentralized and pluralistic society such as Germany, there will always be diverse actors that communicate and inform the general public” [4]. In Germany, these actors include political actors (eg, the Federal Ministry of Health), public health institutes (the RKI at the national level and public health institutes in different federal states and federal districts), federal institutes tasked with public communication (BZgA), a diverse range of web-based and offline media, and individuals (eg, influencers, individual scientists, journalists, politicians, and science communicators). In such an environment, it is particularly important to establish infrastructure for coordinated, professional, and evidence-based health communication. The expert council called for setting up such infrastructure quickly and in a sustainable manner to be better prepared for future crises.

### Aim and Research Questions

At the RKI, much expertise and information are available for social listening and integrated analysis, but they are not fully leveraged to inform risk and health communication. Developing and testing structures to manage the infodemic is in line with the RKI’s strategy and research agenda for the year 2025 [32,33], according to which the institute seeks to develop evidence-based methods for communicating with specific target audiences. To respond to the need for infodemic management in Germany, and specifically at the RKI, in this work, we review and explore opportunities for social listening and integrated analysis to enhance preparedness for future health crises [34,35]. This work focuses on two research questions: At the national public health institute for Germany, the RKI, (1) how can we establish response structures for social listening and integrated analysis? (2) what are the criteria under which these social listening and integrated analyses should be conducted to produce infodemic insights, and the accompanying response structures should be activated? Our case study also aims to serve as a road map for other institutes, within and outside Germany, to follow.

## Methods

### Approach, Aim, and Objectives

On the basis of a desk review, we gathered the available technical guidance [36] and training documentation [28,37-39] on infodemic management, as well as international examples of social listening and insight reporting [24,26,27]. We verified these sources and received technical assistance from our partners, the WHO and the US CDC. We aligned our aim and objectives with the WHO’s public health research agenda for infodemic management [40] stream 1 “Measure and monitor the impact of infodemics during health emergencies.” We aimed to establish a framework for social listening and integrated analysis of public health in the German context. The framework can, in turn, serve as a road map for others to establish infodemic management at other institutions within and outside of Germany. Our key objectives were (1) to identify (potentially) available data sources for social listening and integrated analysis at the RKI, (2) to assess these data sources for their suitability and usefulness for integrated analysis at the RKI, (3) to develop a framework and workflow to combine social listening and integrated analysis, to report back actionable infodemic insights for public health communications by the RKI and stakeholders, and (4) to define criteria for activating infodemic insight reporting in the context of a specific health event or health emergency. The reader should note that the actual insights reporting is outside of the scope of this work.

### Data Sources and (Automation of) Data Extraction

To identify all potential data sources and tools used for social listening in the context of public health, with relevance for Germany, we reviewed the identified documentation (technical documentation [41], [World Health Organization, unpublished data, November 2022], guidance documents, infodemic training materials [28,37-39]), and the methodology of insight reports [24,26,27]. The review team consisted of a health scientist and field epidemiologist (TSB) and a behavioral scientist (CL), both with training in infodemic management [38,42]; a psychology student assistant (PS) who completed the OpenWHO Infodemic Management 101 training [39]; and a data scientist (SW) with specific expertise in web-based social listening using machine learning techniques, including natural language processing (NLP).

First, we reviewed web-based and social media listening tools and analytics, as well as the available social media data sources through application programming interfaces (APIs). We identified the largest social media platforms in Germany for web-based listening based on studies on media consumption [1,43], and identified the respective tools and analytics available for web-based social listening for these platforms. In addition, we reviewed the available APIs of both data aggregators and specific social media platforms based on their (technical) API documentation. Second, we gathered internal, RKI-specific data sources in consultation with colleagues working in the Department of Infectious Disease Epidemiology (including the Emergency Operations Center), Department of Health Monitoring, Risk Communication Unit, Press Office, and Social Media Task Force. Finally, through desk research, we gathered infodemic insight reports by governmental institutions, academia, and nongovernmental organizations.

### Integrated (Data) Analysis to Report Infodemic Insights

Subsequently, we assessed the suitability of these sources for social listening and integrated the analysis of the RKI. Specifically, we assessed how each data source could potentially be analyzed to identify themes and narratives for infodemic insights, how frequently data become updated and available, and the extent to which there may be data protection risks. To best use the available resources at the RKI, we discarded several data sources from the initial list of potential data sources. The initial list of potential data sources and reasons for inclusion and exclusion are presented in Multimedia Appendix 1. Our goal was to gather an as-diverse-as-possible pool of data sources, given limited resources. Tools, analytics, and data sources were ideally open sources and available for noncommercial use, in support of open science, and appropriate for use by public or governmental institutions. For instance, many tools were available to collect and analyze Twitter data, and we initially decided to rely on the freely available tools TweetDeck and epitweetr to cover Twitter data. However, because of recent changes in Twitter, API access (on which these tools rely) now comes with a new cost model [44].

We decided to include all RKI-specific data sources as they reflect questions directed at the RKI that are very different from Twitter data and all infodemic insight data sources that include survey data that are published less frequently as a report and are processed, and thus do not require many additional resources from an RKI-based infodemic insights team (eg, to analyze raw data). These decisions were made within the RKI author group (SB, CL, and PS) after trying out various data sources and tools for practical use, and assessing the costs and benefits of each data source. Thus, the list of data sources presented in this study should be considered as a starting point that covers an as-diverse-set-as-possible, while keeping in mind a reasonable allocation of resources (eg, an initially small team of infodemic managers).

We tabulated the frequency with which each data source generated new data, and thus, the frequency with which it should be analyzed, and the type of data that can be extracted from each data source (ie, different outcome variables or indicators per data source, such as the number of Twitter comments per topic). In addition, we described possibilities within the framework of extending web-based social media listening using recent techniques from the field of NLP. Furthermore, we evaluated each data source based on ethical and data protection considerations (eg, a person writing a private message to the RKI should remain private and not end up in an infodemic insight report). The analysis of the heterogeneous set of identified data sources followed a mixed methods approach to combine qualitative (themes) and quantitative data (analytics). Qualitative data were analyzed through reflexive thematic analysis to identify themes (people’s experiences, views, perceptions, and representations) regarding the public health event of interest [45,46], per data source.

### Data Protection and Ethics

Many different data sources warrant careful consideration regarding privacy and data protection before they can be used for active social listening and integrated analysis. To formally assess data handling, 2 researchers performed an independent risk assessment of each identified data source using the RKI’s standardized data protection questionnaire (version 03/2019). Risk was categorized by the dimensions of low, normal, high, or very high levels of data protection required; potential disagreement was discussed. The RKI’s standardized data protection questionnaire facilitates adherence to the European Union regulation 2016/679 of the European Parliament and of the Council on the protection of natural persons with regard to the processing of personal data and the free movement of such data, and repealing Directive 95/46/EC (General Data Protection Regulation) [47]. Multimedia Appendix 1 is an excerpt of the data protection questionnaire and summarizes what each dimension entails.

### Setting Criteria for Activating Infodemic Insights Reporting

To define criteria for activating infodemic insights reporting structures in the context of a specific health event or health emergency, we (CH and TSB) consulted the RKI’s preparedness and response group. The Emergency Operating Center is within their portfolio, which is situated within the RKI Department for Infectious Disease Epidemiology. We identified and reviewed the RKI’s crisis management structures and preparedness and response plans for Germany and the human resources (including potential surge capacity), to see when and where infodemic response activities could be activated.

## Results

### Identification and Assessment of Data Sources

Table 1 presents the 16 data sources (including tools and reports) that were identified as suitable starting points for web-based and non-web-based social listening and integrated analysis at the RKI, based on the full list of 42 identified sources provided in the Multimedia Appendices 1-3. These fall into 3 main categories: social media and web-based listening, RKI-specific, and infodemic insights. We included primary data sources, such as social media data, requests addressed to the institute (through the Emergency Operations Center [48] and Press Office), task force meetings, press requests, and secondary data sources (ie, secondary research data and reports [24,49-51]). Of note, 3 infodemic insights were COVID-19 specific (ie, COVID-19 Snapshot Monitoring [COSMO] [49], the German Federal Institute for Risk Assessment [BfR]-Corona-Monitor [50], and Ministry of Health COVID-19 digital emergency operating center weekly briefing [52]), the WHO Infodemic Insights reports both on COVID-19 and mpox [24,25], and the general, non-COVID-19 specific reports were published by the Center for Monitoring, Analysis and Strategy (CEMAS) [51].

**Figure 1:**
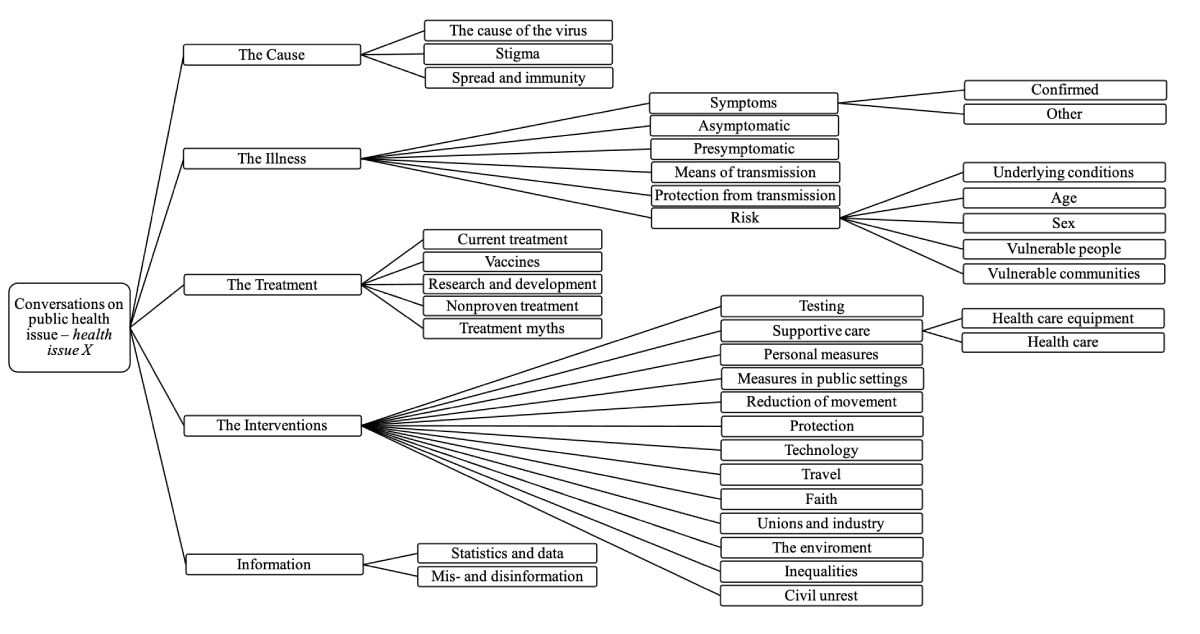
Taxonomy to systematically monitor keywords in conversations related to public health issue X within thematic categories relevant to public health response.

For data extraction, a public health taxonomy helps to identify thematic categories in conversations relevant to the public health response. Validated public health taxonomies for social listening, that is, the COVID-19 and mpox taxonomies developed by the WHO [23,53], formed the basis of the generic taxonomy related to public health issue X, as shown in Figure 1. The taxonomy includes topics and subtopics to capture the breadth of these conversations and help identify the structure and changes in narratives within thematic categories relevant to public health response. To create an infodemic insights report at the RKI, we translated the taxonomy into German (Multimedia Appendix 2). For some data sources, the taxonomy could be directly translated into German and used as Boolean search terms. Question marks can be added to Boolean search terms to identify information voids (questions) [15]. The taxonomy provided for public health issue X is applicable to other infectious diseases or health emergencies and will need to be adapted if the nature of the public health emergency in question is very different from COVID-19 and mpox (eg, war, an extreme weather event). In all events, both the inclusion of data sources and the taxonomy will follow an iterative process and need to be updated regularly to reflect changes in the situation and themes that occur (see the next section).

The German version is provided in Multimedia Appendix 2 based on COVID-19 and mpox taxonomies [23,53].

Several data sources were available weekly (Table 1). Most social media and web-based data (analytics) can be collected more frequently and in real time, but subsequent analyses can still take place weekly. Other data sources can only be monitored less frequently, as surveys and reports are published biweekly, monthly, or on an ad hoc basis.

The data protection risk assessment indicated normal-to low-level risks for social media and web-based listening data sources. RKI-specific data sources were assigned various levels of risk from low to high. Social media activity on RKI accounts was assigned normal (for comments) to high (for direct messages) risk; in all circumstances, user comments or sending a message will remain anonymous. Webpage metrics (RKI website traffic data and search patterns) were considered low risk. The data from the task forces were assigned a normal risk level if RKI employees were informed that the information would be used to develop integrated insights (in anonymized form). emails and phone calls from citizens were assigned a high risk, as these are considered private and nonpublic; however, the data were handled in aggregated form (counts per topic only). RKI press conference questions and Freedom of Information Act requests [54] were considered low risk because they are already in the public domain. So-called “small requests” for information from parliamentary groups or members of the German Federal Parliament [55] were assigned normal risk. Infodemic insights based on research, surveys, and reports from other parties had low risk (anonymous, aggregated data).

**Table 1:** Data sources evaluated based on suitable variables to identify topics and narratives, data availability and analysis frequencies, and data protection risk assessment.

Data source (tool or organizational unit)		Data extraction	Data availability	Data protection risk assessment
**Social media and web-based listening**				
	Data aggregator (eg, Meltwater and Talkwalker)	Data streaming from available social media platforms via API access. Automatically classify posts with taxonomy and count number of posts per taxonomy category (per platform and across platforms). Measure number of interactions (eg, sum of likes or retweets; depending on platform) and change over time. Alternatively, and depending on the aggregator, suitable tools for predefined reporting could be used.	Real time/weekly	Normal
	Twitter (epitweetr [56])	Signal detection (alerts) of an unusual increase in the number of tweets for a specific time place and topic.	Real time/weekly	Normal
	Google searches, web content (Google Alerts)	Published articles, blogs, etc, in a given time frame based on taxonomy and Boolean search strings. Analysis could be automatized with web scraping (access web-based websites and apply taxonomy-based search).	Real time/weekly	Normal
	Google searches (Google Trends)	Compare topic (keywords) to baseline topic (ie, “COVID-19”) and compute relative weekly average. Analysis could be automatized with web scraping (download and analysis of trends data).	Real time/weekly	Low
**Robert Koch Institute-specific**				
	Social media traffic and activity on the RKI’s^a^ social media accounts [57]: [58] (TweetDeck); Mastodon [59]; (Instagram Insights [60]); YouTube [61]; LinkedIn [62]	Count the number of direct messages and comments regarding a topic relative to the overall number of them in the given time frame and questions may be identified to know where information voids exist and new topics may emerge. Instagram specific (Instagram Insights): interactions with posts (likes, number of comments, amount of times post was saved) by topic and the number of people that saw the post (reach). If stories are posted, use the interactions with them by topic relative to the average number of interactions with stories (sum of likes and shares) during the week. Twitter specific (TweetDeck): complementary to automated quantitative data analysis (see above); can be used for qualitative data exploration, that is, to scan trending hashtags regarding COVID-19–related topics.	Weekly	Comments: normal; direct messages: high
	Webpage metrics (RKI website traffic data and search patterns)	Use the number of visits on subpages relative to overall visits on the RKI-web pages.	Weekly	Low
	Task Forces (RKI departments, emergency operations center)	Ask an RKI scientist with technical experience: “What do you think is important and needs to be communicated, now and in the next month?” and let them rank these issues, then create an overall ranking across RKI experts.	Weekly	Normal
	Emails and phone calls from citizens and journalists (RKI press office and Emergency Operations Center)	Emails and calls regarding a topic relative to the overall number of emails and calls in the given time frame and questions may be identified to know where information voids exist and new topics may emerge.	Weekly	High
	RKI press conferences questions	Count the questions regarding a topic during the conference relative to overall number of questions and use them to identify information voids.	Ad hoc, depending on the interval of press conferences	Low
	Freedom of Information Act requests (*FragDenStaat* Portal [54])	Count requests by topics addressed to the RKI.	Ad hoc, when requests are submitted	Low
	“Small requests” from members of the German Federal Parliament [55]	Topics gain insights into the issues that politicians and their constituency are concerned with.	Ad hoc, when requests are submitted	Normal
**Infodemic insights**				
	COSMO^b^ Snapshot monitoring [49]	Scan report for sections relevant to the taxonomy. The output depends on how questions are framed (eg, “How informed do you feel about vaccinations?” “X % do not feel very informed”). Read the summary: some results about knowledge show information voids. This also depends on how the question was framed in the survey. For relevant questions, use the proportion of respondents not knowing or worrying. COSMO could also be used for identifying information voids and new topics that need communication.	Ad hoc, when report is published (currently biweekly)	Low
	BfR^c^-Corona-Monitor [50]	Perceived informedness: how informed do you feel regarding topic X? (less informed—more important topic). Use the proportion of respondents not feeling informed by topic. This may be used to identify information voids and new topics that emerge and need communication.	Ad hoc, when report is published (biweekly/monthly)	Low
	MoH^d^ COVID-19 Digital Emergency Operations Center, weekly briefing [52] (Cosmonauts and Kings)	Browse reports for sections relevant to the taxonomy. The report summarizes findings of existing studies, social media data (posts, comments, analytics), and reports misinformation (Telegram, fact-checking organizations), including narratives regarding the MoH.	Weekly	Low
	WHO^e^ Infodemic Insights report [24,25] (Marble Global)	Browse report sections relevant to the taxonomy (questions in English-speaking communities may also be relevant to German-speaking communities; misinformation across countries may be similar).	Weekly	Low
	CEMAS^f^ [51]	Browse reports for sections relevant to the taxonomy. The output depends on how questions are framed. Scan reports and blog posts for relevant topics.	Ad hoc, when report is published	Low

^a^RKI: Robert Koch Institute.

^b^COSMO: COVID-19 Snapshot Monitoring.

^c^BfR: German Federal Institute for Risk Assessment (Bundesinstitut für Risikobewertung).

^d^MoH: Ministry of Health.

^e^WHO: World Health Organization.

^f^CEMAS: Center for Monitoring, Analysis and Strategy.

### Automation of Data Extraction and Analysis From Web-Based Social Media

Web-based social media platforms are constantly producing large amounts of data. For instance, approximately 30 million German tweets were sent every month (not shown, based on an analysis from the Twitter API [44]). Ideally, data analysis should be able to cope with such data streams to derive results that represent the entire data set in a timely manner. In the context of this framework, this can be achieved by collecting data through APIs and deriving quantitative metrics automatically (ie, the number of likes). Typically, APIs offer a type of keyword-based search, allowing the incorporation of taxonomy directly into data collection. The size of data and availability of APIs makes web-based social media listening well-suited for automatization, freeing team members to spend more time on qualitative or more involving data analysis.

Importantly, this lays the foundation for systems with more complex analytical methods, as modern data-driven systems are typically built as automated pipelines (from data collection to analysis). Technological advancements of the last years in AI, particularly in NLP, have led to a wide range of new approaches across different domains, including public health, for text and language analysis [63]. A prominent technique is sentiment analysis, which aims to determine the sentiment expressed in texts, for example, toward vaccination [64]. Furthermore, recent approaches in topic modeling (eg, BERTopic [65]) have demonstrated the ability of language models to cluster texts (such as social media posts) into semantically coherent groups (topics) in large text corpora. These latent topics are usually not known or assumed a priori, and such approaches can help to find unexpected topics in social media conversations. Consequently, data-driven approaches may allow the automatic derivation of an adaptive taxonomy from data that allows topics to emerge over time. This differs from a domain-driven taxonomy (Figure 1), which is built a posteriori and based on the domain knowledge of the involved researchers. Although data-driven approaches offer interesting possibilities, they may be challenging to operate as they require specific technical knowledge (eg, machine learning) and special computer hardware (eg, GPU). In addition, incomprehensible results generated by language models in NLP applications are generally difficult to interpret and require expert knowledge. By contrast, the proposed domain-driven taxonomy (Figure 1) is technically easy to implement and to understand, facilitate, and enable the use of teams with different levels of technical know-how in health institutes.

### Integrated Analysis and Workflow to Report Infodemic Insights

**Figure 2:**
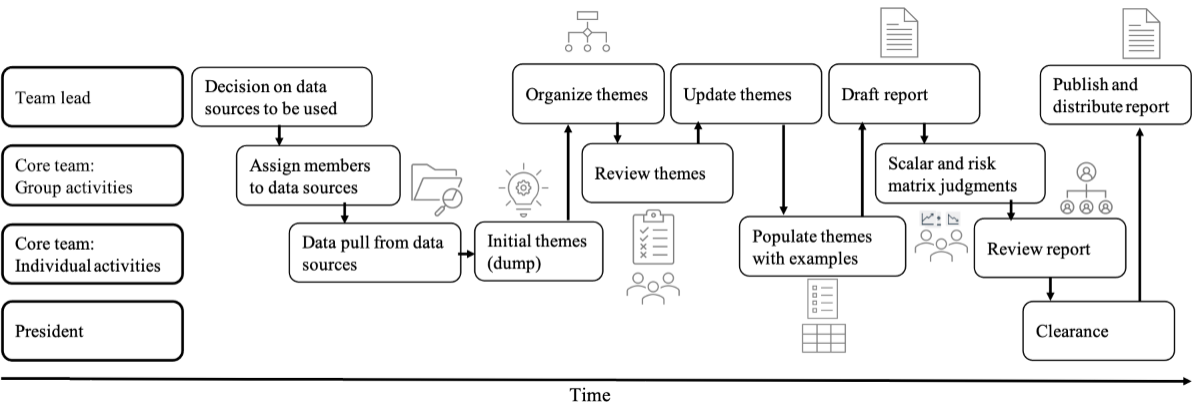
Swim lane graph showing roles and responsibilities across the infodemic management team, as well as a proposed workflow to combine different data sources into an infodemic insights report. Adapted from Kolis and Voegeli [66].

The taxonomy (Figure 1) serves as a starting point for deductively coding themes that may emerge in the data and ensures basic comparability across data sources. Additional topics not contained in the predefined taxonomy are inductively coded and can be added if they are not contained in the predefined taxonomy. Topics that emerge across many different data sources may be (relatively) more important than topics that emerge only rarely; however, final judgments of the relative importance of particular topics and the urgency with which they require a response are made based on a risk matrix. Figure 2 shows the proposed workflow for creating an infodemic insight report based on the data sources listed in Table 1. The infodemic insights team lead decides which data sources should be included for analysis based on the availability of the team members and the timeframe. Each team member can be responsible for one or more data sources and independently extract the data and identify themes and topics based on the taxonomy (Figure 1) and add additional themes that emerge, collected in a spreadsheet. In a group meeting, the core team discusses the initial data extraction and identifies potential examples of insights that could be included in the report as illustrative examples of the identified narrative (eg, public comments or posts on Twitter).

**Figure 3:**
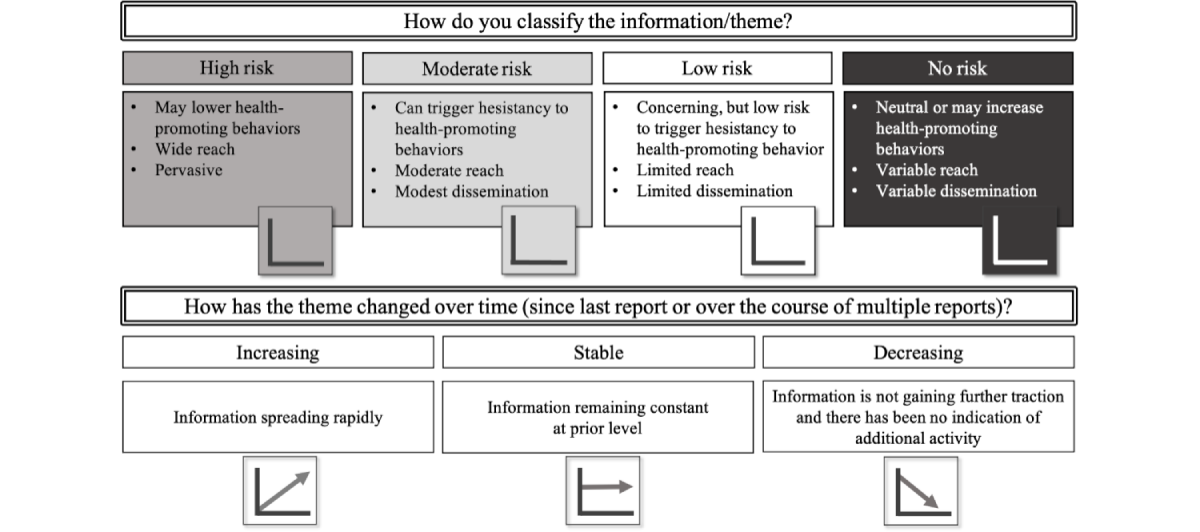
Risk matrix and scalar judgments, adapted from the US Centers for Disease Control and Prevention Vaccine Confidence Report methodology [26,27].

The team lead drafts an insight report based on the main themes and topics. The core team then judges each theme in a risk matrix to determine the risk level for each theme, determine which themes to prioritize to be included in the report, and uses a scalar judgment (Figure 3), which is based on the US CDC’s Vaccine Confidence Insights Reports [26,27] and adapted for broader public health topics such as chronic diseases, natural disasters, or other emergency responses (such as mpox [53]). The risk matrix is a classic decision matrix, where the first axis is the degree of impact on the uptake of a health-promoting behavior, and the second axis is the frequency it appears in the data sources relative to previous data collection points. High-risk themes can be those that lower health-promoting behaviors, have wide reach, and are pervasive, whereas low-risk themes are concerning, but have limited reach and dissemination. Moderate risk can trigger hesitancy to follow health-promoting behaviors, tend to have moderate reach, and moderate dissemination. Low risk is assigned to themes that can trigger hesitancy but have limited reach and dissemination. No risk themes can include themes that do not concern or even increase health-promoting behaviors. Subsequently, scalar judgment assesses the directionality of the theme over time (eg, since the last report): increasing, stable, and decreasing. Then, the entire team reviews the report, which then undergoes scientific clearance by the RKI’s president and is distributed to our stakeholders.

### Reporting Back Actionable Infodemic Insights

Communicating infodemic insights and actionable recommendations based on social listening and integrated analysis is essential to support the public health response. The level of reporting details depends on the availability of resources (ie, the number of team members available and the number of hours that can be spent on the project). The output could range from a full-fledged insights report, including actionable recommendations, to a potential set of indicators that can be integrated into existing reports (eg, the RKI’s situation reports focused on epidemiological trends and developments). In public health emergencies, speed trump perfection, and depending on the situation, a quick overview in an (epidemiological) situation report may trump a stand-alone (infodemic) report. Nevertheless, careful consideration of the impact of the published report or indicator is needed before it is sent to various audiences or published on the web. To put the integrated analysis results to best use, the infodemic insights report should be shared widely with partners and interested stakeholders who can use these insights for risk communication and community engagement activities. These partners and stakeholders include, but are not limited to, other German public health institutes (state and local), governmental institutions and ministries (Ministry of Health and Federal Centre of Health Education), community and religious organizations, science communicators, journalists (media), and fact-checking organizations.

### Criteria for Activating Social Listening and Integrated Analysis Structures

Social listening and integrated analysis structures can be activated in the context of the RKI’s crisis management structures [48,67]. Owing to the primary responsibility at the district and federal state levels in dealing with important epidemic situations, the RKI (federal level) usually only becomes active in the case of major or exceptional epidemiological situations [67]. The term important epidemic situations refers to either the local or temporal clustering of threatening communicable diseases, threatening diseases in which pathogens or toxins can be considered as the cause, or the concretely justified possibility that such diseases or illnesses may occur in the near future [67]. The activation of crisis management structures depends on the internal evaluation of the internal workload, number of possibly affected people, disease severity, geographic distribution, and public perception of the situation [48]. However, social listening and integrated analysis structures to report infodemic insights can also be activated for public health emergencies concerning Europe, as a support and prevention of the spread of a communicable disease to Germany, as judged by RKI experts, as our analyses focus on the German-speaking context.

Editable versions of Figures 1-3 and Multimedia Appendix 2 are available in Multimedia Appendix 4.

## Discussion

### Principal Findings

In this study, we propose a framework to establish social listening and integrated analysis to report infodemic insights at the National Public Health Institute in Germany. We identified and assessed 16 different types of data sources for social listening (at the time of writing, fall or winter 2022/2023) that fall into 3 main categories: social media and web-based listening data, RKI-specific data, and infodemic insights. Monitoring these web-based and non-web-based data sources can help to understand the population’s understanding, perceptions, concerns and questions, information voids, narratives, misinformation and disinformation, and other relevant information about people’s reactions to a health topic in Germany [24]. Most of these data sources can be analyzed weekly to detect current trends and narratives and to inform a timely response. Emerging data sources can also be included. One forthcoming data source that has the potential to provide key infodemic insights is the platform “RKI Panel—Health in Germany” [67], which plans to repeatedly survey a group of people on various health science topics. Social media and web-based listening data sources are available through different channels such as APIs, commercial data aggregators, or through manual searches. Consequently, obtaining and processing a comprehensive data set is a nontrivial task and is related to both the computational resources and available funds. For example, in the case of web-based social listening, the cost of using a commercially available data aggregator should be weighed against the technical expertise needed to collect and manage data from multiple freely or commercially available sources (ie, social media platforms; Multimedia Appendices 1-3). The selection of data sources used for each public health event might differ, depending on the situation and resources available.

Subsequently, a methodological examination was conducted to produce infodemic insights for the RKI. These insights can point out confusion, where the health authority is experiencing communication failures with the public, and what policy or programmatic levers can be used to address it (including but not limited to risk communication activities). Although there are many reasons for misinformation spreading [68] (eg, individual differences, information voids), identifying and tracking misinformation early can help with prebunking and debunking misinformation. For guidance on when and how to prebunk and debunk, see the Debunking Handbook [11].

The scope and extent of the integrated analysis that is put into place depends on the resources available to the project. We relied on prior experiences by the US CDC [26] to lay out the resources needed for different tasks and responsibilities, such as analyzing specific data sources, identifying common themes across data sources, and finally writing up a structured insight report. The outputs are flexible: either key infodemic insights are added to existing situation reports or a stand-alone report can be published. The primary audience for the infodemic insights reports is the RKI Emergency Operations Center and task forces. In addition, other public stakeholders and communicators involved in acute public health events [67], including but not limited to the Federal Ministry of Health, the Federal Centre of Health Education, the Federal Institute for Risk Assessment, and state- and local-level public health authorities and governmental institutions, could benefit from these reports. Collaboration and exchange with these organizations should be sustained and strengthened through wide sharing of infodemic insights and could also create access to additional data sources for social listening (eg, analysis of hotlines for citizens from the Federal Centre of Health Education).

Finally, we considered different criteria for activating integrated analysis structures and described how these activities could fit into the RKI’s existing crisis response structures and Germany’s legal framework [48,67]. The infodemic management activities proposed in this work are deemed suitable for addition to the existing preparedness and response structures at the RKI.

As we applied the methods of the WHO’s infodemic insights report to the German context on the methodological level, this provides an opportunity to test how robust findings are across languages and geography (eg, compared with findings in the context of the WHO Infodemic Insights report). It is important to note that German speaking does not mean “within Germany, ” as netizens are widely connected. There is both a German-speaking community outside Germany, Austria, and Switzerland (the DACH [Germany—D, Austria—A, and Switzerland—CH] region) that would be captured well by the analysis, and a non-German-speaking community within Germany that would not be captured well by the proposed analysis. Moreover, our case study for the German context also serves as a roadmap to establish infodemic management at other institutes, both within and outside of Germany.

### Limitations

Thus, the proposed activities should be interpreted carefully. The identified data sources include more web-based than non-web-based sources, and all data sources cover different audiences and come with inherent biases. Twitter appears to be a particularly fruitful source, as the data available for analysis are very comprehensive [69]. However, the future of Twitter API for academic research access is uncertain [65]. Moreover, despite being a popular platform, Twitter users are not representative of the general population [67]. Twitter has a major influence on the information ecosystem, for example, through journalists who can bring trending topics to offline media or scientists and politicians who serve as multipliers. Furthermore, not all key social media data sources have API. However, the overall direction points toward open social media data as further social media channels have recently implemented research APIs (eg, TikTok and YouTube; see Multimedia Appendices 1-3). However, the use regulations for these research accesses vary widely in terms of their eligibility. For instance, TikTok’s new research program is currently only available for US-based research, and the YouTube research program is only eligible for researchers from higher education institutions (that can grant degrees). These regulations limit the use of research programs for public and governmental institutions, such as the RKI. Data access may only be available via commercial options, either directly from social media platforms or data aggregators.

It is still necessary to include more offline sources such as community dipstick surveys or town hall discussions. This would require additional personnel trained in conducting field studies (eg, anthropology and ethnography). Similarly, we included citizen questions directed to the RKI but not to other public institutions (such as the Federal Ministry of Health or the Federal Centre for Health Education), science communicators, politicians, or other actors. Importantly, even though the public seeks information at the RKI, the RKI predominantly deals with (public) health professionals, which could affect data collection for social listening activities. Public health professionals can, however, still provide valuable insights into ongoing narratives in the general population and serve as an audience for the insights report. Furthermore, there is a trade-off between speed and accuracy. The goal of an integrated analysis is to identify important narratives quickly and respond rapidly (eg, to misinformation). Iterative updates, internal (clearance) procedures, and publishing timeframes can hinder swift publication of infodemic insights. Even ambitious weekly or biweekly reporting may be too slow for a timely operational response to the current narrative, information voids, or an outbreak of misinformation, especially on social media.

### Next Steps

To put the proposed framework for social listening and integrated analysis into practice [10], several activities were planned to operationalize social listening and integrated analysis to report infodemic insights at the RKI. First, the proposed setup for data handling will be submitted for ethics and data protection clearance. For data protection clearance, the identified data sources and variables to be obtained are discussed closely with the data protection officer. Second, in collaboration with the RKI’s newly established Centre for Artificial Intelligence in Public Health Research, we will seek to further explore the RKI’s web-based social listening capacities using artificial intelligence techniques and the data sources identified for social media and web-based listening. Third, the integrated analysis proposed here could potentially be piloted in the form of a field infodemiology project by field epidemiology fellows in Germany, under the supervision of and in collaboration with the RKI’s risk communication group and the Department of Infectious Disease Epidemiology, Unit for Preparedness and Response. During this field phase (pilot), the data sources, taxonomy, integrated analysis, and workflow were tested and evaluated in the German context. This will help identify potential difficulties in combining different data sources and in subsequent reporting, particularly as many decisions in the process are subjective. The pilot will also provide insight into the amount of (human) resources needed to operationalize the proposed social listening and integrated analysis activities and their appropriate turnaround and reporting time frame. If the pilot is successful, the analysis can be extended to other health topics (eg, climate crisis). Therefore, novel taxonomies and Boolean search strings need to be developed. The need to constantly analyze narratives surrounding a particular topic (and which) needs to be evaluated and re-evaluated.

Moreover, a continuous and iterative evaluation and re-evaluation of the data sources, infodemic insights reporting, and workflow is required to build sustainable and effective infodemic management activities at the RKI. International exchanges with other public health institutes building experience with social listening [70,71] and communities of practice can foster further advancement in this area. A forthcoming guidance on developing infodemic insight reports will be published in a manual by the WHO and its partners [72]. A final important next step is to involve stakeholders and partners and create appreciation and demand for infodemic insights reporting and integrate this into regular policy making and programmatic decision-making [10]. Actively reaching out to these partners is essential for creating a demand for the report. Conversely, these partners could deliver additional data sources and inputs for future studies. Ultimately, an English version of the findings could be reported to the ECDC and WHO to add to the European and global level of reporting on the infodemic (eg, national surveillance data are being shared through this route, feeding into international surveillance reports).

### Conclusions

The RKI identified and assessed a wide range of data sources for social listening and integrated analysis to report actionable infodemic insights, ensuring a valuable first step in establishing and operationalizing infodemic management at the RKI. Setting up the right tools for social media and web-based social listening will help to automate parts of the process. Piloting the proposed work will help refine the proposed workflow and show its value in informing the public health response. Ultimately, this work will provide better and targeted public health communication at the RKI and beyond.

## Appendix

Multimedia Appendix 1 Risk assessment of data protection requirements, based on the data protection questionnaire for new procedures for processing personal data at the Robert Koch Institute (Version 03/2019).

Multimedia Appendix 2 Taxonomy to systematically monitor keywords in conversations related to public health issue X within thematic categories relevant to public health responses.

Multimedia Appendix 3 Full list of in- and exclusions of 42 potential data sources for social listening.

Multimedia Appendix 4 Editable versions of Figures 1-3 and Multimedia Appendix 2.

## Abbreviations

AI: artificial intelligence
API: application programming interface
BZgA: Bundeszentrale für gesundheitliche Aufklärung
CEMAS: Center for Monitoring, Analysis and Strategy
ECDC: European Centre for Disease Control and Prevention
NLP: natural language processing
OECD: Organisation for Economic Co-operation and Development
RKI: Robert Koch Institute
US CDC: United States Centers for Disease Control and Prevention
WHO: World Health Organization

## References

[1] Newman N, Fletcher R, Robertson CT, Eddy K, Nielsen RK (2022). Reuters Institute Digital News Report 2022. Reuters Institute for the Study of Journalism.

[2] Wardle C, Derakhshan H (2017). Information disorder: toward an interdisciplinary framework for research and policy making. Council of Europe report.

[3] Briand SC, Cinelli M, Nguyen T, Lewis R, Prybylski D, Valensise CM, Colizza V, Tozzi AE, Perra N, Baronchelli A, Tizzoni M, Zollo F, Scala A, Purnat T, Czerniak C, Kucharski AJ, Tshangela A, Zhou L, Quattrociocchi W (2021). Infodemics: a new challenge for public health. Cell.

[4] (2022). 5. Stellungnahme des ExpertInnenrates der Bundesregierung zu COVID-19 - Zur Notwendigkeit evidenzbasierter Risiko- und Gesundheitskommunikation. Informationsamt der Bundesregierung.

[5] Habersaat KB, Betsch C, Danchin M, Sunstein CR, Böhm R, Falk A, Brewer NT, Omer SB, Scherzer M, Sah S, Fischer EF, Scheel AE, Fancourt D, Kitayama S, Dubé E, Leask J, Dutta M, MacDonald NE, Temkina A, Lieberoth A, Jackson M, Lewandowsky S, Seale H, Fietje N, Schmid P, Gelfand M, Korn L, Eitze S, Felgendreff L, Sprengholz P, Salvi C, Butler R (2020). Ten considerations for effectively managing the COVID-19 transition. Nat Hum Behav.

[6] Loss J, Boklage E, Jordan S, Jenny MA, Weishaar H, El Bcheraoui C (2021). Risk communication in the containment of the COVID-19 pandemic: challenges and promising approaches. Bundesgesundheitsblatt Gesundheitsforschung Gesundheitsschutz.

[7] COVID-19 National Preparedness Collaborators (2022). Pandemic preparedness and COVID-19: an exploratory analysis of infection and fatality rates, and contextual factors associated with preparedness in 177 countries, from Jan 1, 2020, to Sept 30, 2021. Lancet.

[8] Savoia E, Piltch-Loeb R, Masterson E, Testa MA, Fantini MP, Tsolova S (2022). Rapid analysis of the first year of the COVID-19 pandemic response for the development of preparedness measures for public communication. SSRN J.

[9] OECD (2022). Building Trust to Reinforce Democracy: Main Findings from the 2021 OECD Survey on Drivers of Trust in Public Institutions. Organisation for Economic Cooperation and Development.

[10] (2022). WHO policy brief: COVID-19 infodemic management. World Health Organization.

[11] Lewandowsky S, Cook J, Ecker U, Albarracín D, Amazeen MA, Kendeou P, Lombardi D, Newman EJ, Pennycook G, Porter E, Rand DG, Rapp DN, Reifler J, Roozenbeek J, Schmid P, Seifert CM, Sinatra GM, Swire-Thompson B, van der Linden S, Vraga EK, Wood TJ, Zaragoza MS (2020). The Debunking Handbook 2020. Center For Climate Change Communication.

[12] Herzog SM, Hertwig R (2019). Kompetenzen mit "Boosts" stärken: Verhaltenswissenschaftliche Erkenntnisse jenseits von "Nudging". Appl Psychol.

[13] Eysenbach G (2008). Eysenbach: infodemiology and infoveillance. SlideShare.

[14] Eysenbach G (2009). Infodemiology and infoveillance: framework for an emerging set of public health informatics methods to analyze search, communication and publication behavior on the Internet. J Med Internet Res.

[15] Tangcharoensathien V, Calleja N, Nguyen T, Purnat T, D'Agostino M, Garcia-Saiso S, Landry M, Rashidian A, Hamilton C, AbdAllah A, Ghiga I, Hill A, Hougendobler D, van Andel J, Nunn M, Brooks I, Sacco PL, De Domenico M, Mai P, Gruzd A, Alaphilippe A, Briand S (2020). Framework for managing the COVID-19 infodemic: methods and results of an online, crowdsourced WHO technical consultation. J Med Internet Res.

[16] Eysenbach G (2020). How to fight an infodemic: the four pillars of infodemic management. J Med Internet Res.

[17] Scales D, Gorman J, Jamieson KH (2021). The COVID-19 infodemic - applying the epidemiologic model to counter misinformation. N Engl J Med.

[18] (2022). Core competencies in applied infectious disease epidemiology in Europe. European Centre for Disease Prevention and Control.

[19] Gesser-Edelsburg A (2021). Using narrative evidence to convey health information on social media: the case of COVID-19. J Med Internet Res.

[20] (2021). Finding the signal through the noise: a landscape review and framework to enhance the effective use of digital social listening for immunisation demand generation. Gavi, UNICEF, WHO, Vaccine Demand Hub, HealthEnabled.

[21] Newberry C, Macready H (2022). What is Social Listening, Why it Matters + 14 Tools to Help. Hootsuite Inc.

[22] Purnat TD, Wilson H, Nguyen T, Briand S (2021). EARS - a WHO platform for AI-supported real-time online social listening of COVID-19 conversations. Stud Health Technol Inform.

[23] Purnat TD, Vacca P, Czerniak C, Ball S, Burzo S, Zecchin T, Wright A, Bezbaruah S, Tanggol F, Dubé È, Labbé F, Dionne M, Lamichhane J, Mahajan A, Briand S, Nguyen T (2021). Infodemic signal detection during the COVID-19 pandemic: development of a methodology for identifying potential information voids in online conversations. JMIR Infodemiology.

[24] Purnat TD, Nguyen T, Ishizumi A, Yau B, White B, Cecchini S, Samuel R, Hess S, Bezbaruah S, Briand S (2022). Delivering actionable infodemic insights and recommendations for the COVID-19 pandemic response. Wkly Epidemiol Rec.

[25] (2022). Monkeypox Infodemic Insights Report.

[26] (2022). COVID-19 state of vaccine confidence insights reports. Centers for Disease Control and Prevention.

[27] (2022). CDC’s monkeypox state of vaccine confidence insights report. Centers for Disease Control and Prevention.

[28] Purnat TD, Vacca P, Burzo S, Zecchin T, Wright A, Briand S, Nguyen T (2021). WHO digital intelligence analysis for tracking narratives and information voids in the COVID-19 infodemic. Stud Health Technol Inform.

[29] (2022). Monkeypox multi-country outbreak - first update. European Centre for Disease Prevention and Control.

[30] (2022). Infodemic. World Health Organization.

[31] The Expert Council of the Federal Government. Die Bundesregierung.

[32] (2017). Robert Koch institute 2025 strategy (RKI 2025): promoting research and evidence, sharing knowledge, protecting and improving health. Robert Koch institute.

[33] (2019). RKI 2018-2025 summary research agenda. Robert Koch Institute.

[34] (2021). WHO competency framework: building a response workforce to manage infodemics. World Health Organization.

[35] Rubinelli S, Purnat TD, Wilhelm E, Traicoff D, Namageyo-Funa A, Thomson A, Wardle C, Lamichhane J, Briand S, Nguyen T (2022). WHO competency framework for health authorities and institutions to manage infodemics: its development and features. Hum Resour Health.

[36] World Health Organization Regional Office for Europe (2021). Monitoring wider effects of the COVID-19 pandemic. A Webinar to Learn About Monitoring the Wider Effects of the COVID-19 Pandemic.

[37] World Health Organization, US Centers for Disease Control and Prevention, Africa Centres for Disease Control and Prevention, Risk communication and community engagement collective service, First Draft (2020). First WHO infodemic manager training. World Health Organization.

[38] US Centers for Disease Control and Prevention, ECDC, UNICEF partners (2021). Second WHO infodemic manager training. World Health Organization.

[39] (2022). Infodemic management 101. World Health Organization.

[40] (2021). WHO public health research agenda for managing infodemics. World Health Organization.

[41] (2022). Advancing infodemic management in risk communication and community engagement in the WHO European Region: implementation guidance. World Health Organization, Europe.

[42] US Centers for Disease Control and Prevention, UNICEF, RCCE collective service (2022). 3rd WHO infodemic manager training. World Health Organization.

[43] Leuker C, Hertwig R, Gumenik K, Eggeling LM, Hechtlinger S, Kozyreva A, Samaan L, Fleischhut N (2020). Wie informiert sich die Bevölkerung in Deutschland rund um das Coronavirus? Umfrage zu vorherrschenden Themen und Gründen, dem Umgang mit Fehlinformationen, sowie der Risikowahrnehmung und dem Wissen der Bevölkerung rund um das Coronavirus. Max-Planck-Institut für Bildungsforschung.

[44] (2023). Twitter API access levels and versions. Twitter Developer Platform.

[45] Spencer L, Ritchie J, O'Connor W, Ritchie J, Lewis J (2003). Analysis: practices, principles and processes. Qualitative Research Practice: A Guide for Social Science Students and Researchers.

[46] Braun V, Clarke V (2019). Reflecting on reflexive thematic analysis. Qual Res Sport Exerc Health.

[47] (2016). Regulation (EU) 2016/679 of the European Parliament and of the Council of 27 April 2016 on the protection of natural persons with regard to the processing of personal data and on the free movement of such data, and repealing Directive 95/46/EC. Official Journal of the European Union.

[48] Halm A, Grote U, An der Heiden M, Hamouda O, Schaade L, Rexroth U, RKI-Lagezentrums-Gruppe (2021). Crisis management at the Robert Koch Institute during the COVID-19 pandemic and the exchange between federal and state governments. Bundesgesundheitsblatt Gesundheitsforschung Gesundheitsschutz.

[49] WHO Regional Office for Europe (2020). COVID-19 Snapshot Monitoring (COSMO Standard): monitoring knowledge, risk perceptions, preventive behaviours, and public trust in the current coronavirus outbreak - WHO standard protocol. PsychArchives.

[50] (2022). BfR-Corona-Monitor. Bundesinstitut für Risikobewertung.

[51] CeMAS: Center for Monitoring, Analysis, and Strategy.

[52] (2022). Ministy of Health COVID-19 digital emergeny operating centre, weekly briefing Digitales Lagezentrum - Wöchentliches Briefing.

[53] (2022). Public health taxonomy for social listening on monkeypox conversations: for use in infodemic monitoring and insights generation. World Health Organization, Epidemic and Pandemic Preparedness and Prevention Team.

[54] Requests to Robert Koch-Institut. FragDenStaat.

[55] Kleine Anfrage. Deutscher Bundestag.

[56] Espinosa L, Wijermans A, Orchard F, Höhle M, Czernichow T, Coletti P, Hermans L, Faes C, Kissling E, Mollet T (2022). Epitweetr: early warning of public health threats using Twitter data. Euro Surveill.

[57] Soziale Medien, Newsletter und RSS-Feeds. Robert Koch-Institut.

[58] Robert Koch-Institut. Twitter.

[59] Robert Koch-Institut. Mastodon.

[60] Robert Koch-Institut. Instagram.

[61] Robert Koch-Institut. YouTube.

[62] Robert Koch Institute. Linkedin.

[63] Baclic O, Tunis M, Young K, Doan C, Swerdfeger H, Schonfeld J (2020). Challenges and opportunities for public health made possible by advances in natural language processing. Can Commun Dis Rep.

[64] Niu Q, Liu J, Kato M, Shinohara Y, Matsumura N, Aoyama T, Nagai-Tanima M (2022). Public opinion and sentiment before and at the beginning of COVID-19 vaccinations in Japan: Twitter analysis. JMIR Infodemiology.

[65] Grootendorst M BERTopic: neural topic modeling with a class-based TF-IDF procedure. arXiv.

[66] Kolis J, Voegeli C (2021). Integrated analysis of the iceberg. Proceedings of the 5th Virtual WHO Infodemic Management Conference.

[67] (2013). Allgemeine Verwaltungsvorschrift über die Koordinierung des Infektionsschutzes in epidemisch bedeutsamen Fällen (Verwaltungsvorschrift-IfSG-Koordinierung - IfSGKoordinierungs-VwV). Die Bundesregierung.

[68] Chan MP, Jones CR, Hall Jamieson K, Albarracín D (2017). Debunking: a meta-analysis of the psychological efficacy of messages countering misinformation. Psychol Sci.

[69] Twitter API - academic research access. Twitter Developer Platform.

[70] Lohiniva A, Sane J, Sibenberg K, Puumalainen T, Salminen M (2020). Understanding coronavirus disease (COVID-19) risk perceptions among the public to enhance risk communication efforts: a practical approach for outbreaks, Finland, February 2020. Euro Surveill.

[71] Lohiniva AL, Sibenberg K, Austero S, Skogberg N (2022). Social listening to enhance access to appropriate pandemic information among culturally diverse populations: case study from Finland. JMIR Infodemiology.

[72] (2023). How to develop an infodemic insights report in 6 steps. World Health Organization.

