# Supplementary material for: Establishing Infodemic Management in Germany: A Framework for Social Listening and Integrated Analysis to Report Infodemic Insights at the National Public Health Institute
Source: JMIR Infodemiology. 2023 Jun 1;3:e43646. doi: 10.2196/43646 (PMC10273031; doi:10.2196/43646)
Supplement: Multimedia Appendix 1 [file infodemiology_v3i1e43646_app1.pdf]

Multimedia Appendix 1.

Supplemental Table 1. Risk assessment of data protection requirement, based on the data protection questionnaire for new procedures for processing of personal data at the Robert Koch Institute (Version 03/2019).

**Protection requirement**	**Description**
Low	Anonymous data
Normal	Since any processing of personal data constitutes an interference with the fundamental rights of the data subject, the need for protection can never be lower than "normal." Consequently, only processing operations involving non-personal, i. e. anonymous, data can be less in need of protection.
High	The following processing scenarios, listed as examples, imply an intensity of intervention that may result in a higher than normal need for protection:<br>- Processing of unchangeable personal data that can serve as an anchor for profiling for a lifetime or that can be assigned (e.g., biometric data, genetic data),<br>- Dissemination of uniquely identifying, highly linkable data (e.g., lifetime health insurance number, tax ID),<br>- Processing of data with potential impact on the image/reputation of the data subject,<br>- Processing of data in a procedure with potential impact on the physical integrity of the data subject,<br>- Processing of data that can realistically be expected to have an impact on the exercise of fundamental rights of a large number of data subjects (e.g., increasingly widespread, public video surveillance),<br>- Risk of discrimination, stigmatization (e.g., becoming aware of HIV infection),<br>- Interference with particularly protected internal sphere of life of a data subject (e.g. sexual behaviour),<br>- Personal health data
Very high	A very high need for protection is to be assumed if a person affected is directly dependent on the decisions or services of the organization for his or her existence and additional risks for the person affected are not noticeable.
