# Supplementary material for: Establishing Infodemic Management in Germany: A Framework for Social Listening and Integrated Analysis to Report Infodemic Insights at the National Public Health Institute
Source: JMIR Infodemiology. 2023 Jun 1;3:e43646. doi: 10.2196/43646 (PMC10273031; doi:10.2196/43646)

**Multimedia Appendix 2.**

**Supplemental Figure 1. Taxonomy to systematically monitor keywords in conversations related to public health issue X within thematic categories relevant to public health responses.**
(German version; English version as Figure 1).

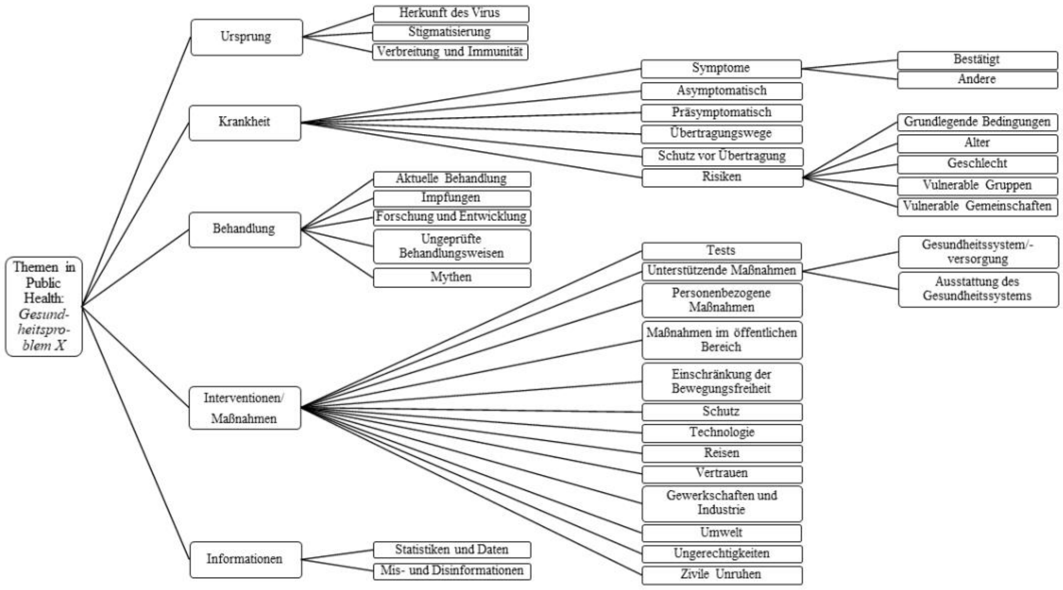

Supplement: Multimedia Appendix 2 [file infodemiology_v3i1e43646_app2.pdf]
