# Supplementary material for: Establishing Infodemic Management in Germany: A Framework for Social Listening and Integrated Analysis to Report Infodemic Insights at the National Public Health Institute
Source: JMIR Infodemiology. 2023 Jun 1;3:e43646. doi: 10.2196/43646 (PMC10273031; doi:10.2196/43646)

## Slide 1
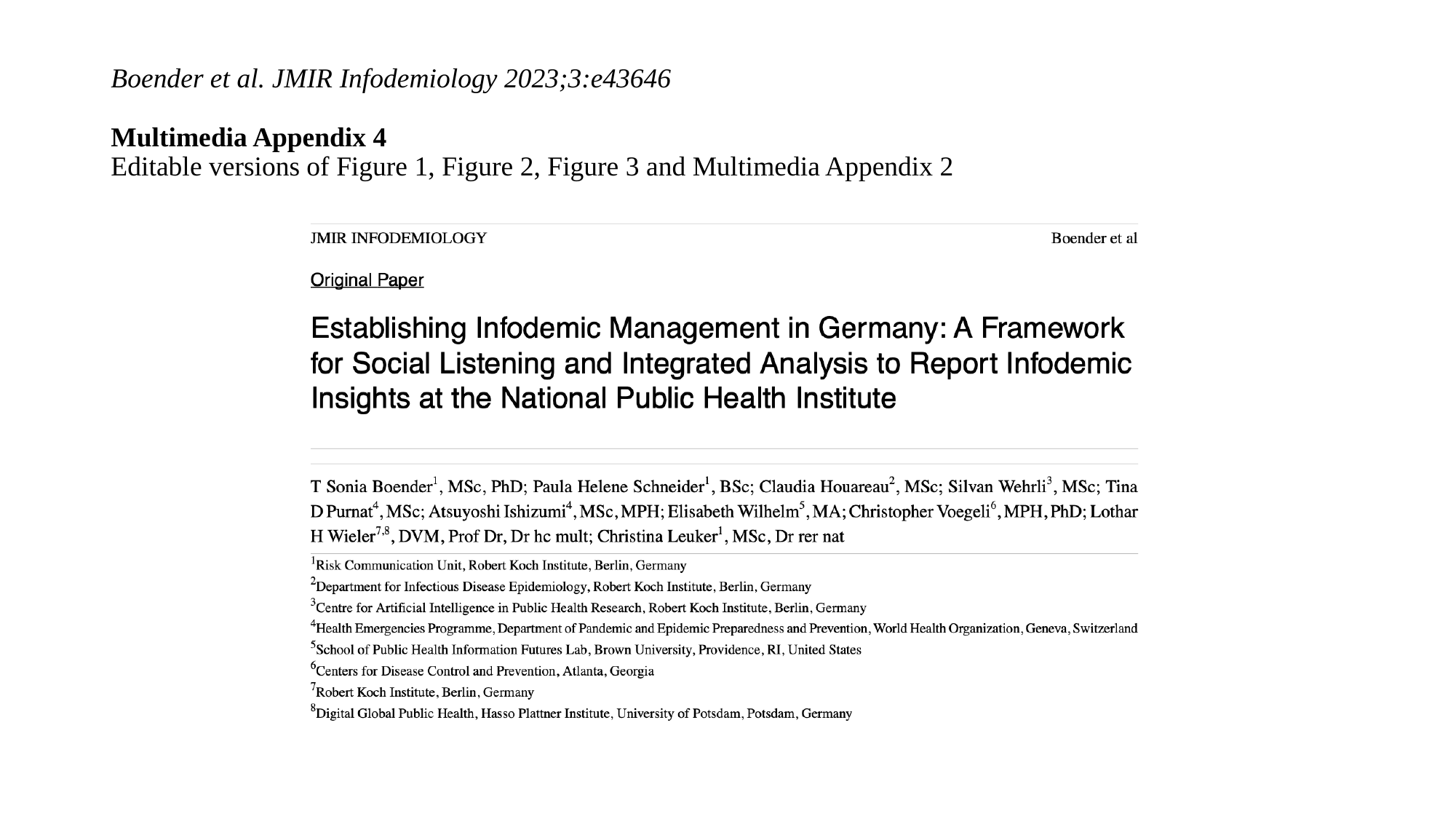

# Boender et al. JMIR Infodemiology 2023;3:e43646 Multimedia Appendix 4 Editable versions of Figure 1, Figure 2, Figure 3 and Multimedia Appendix 2

## Slide 2
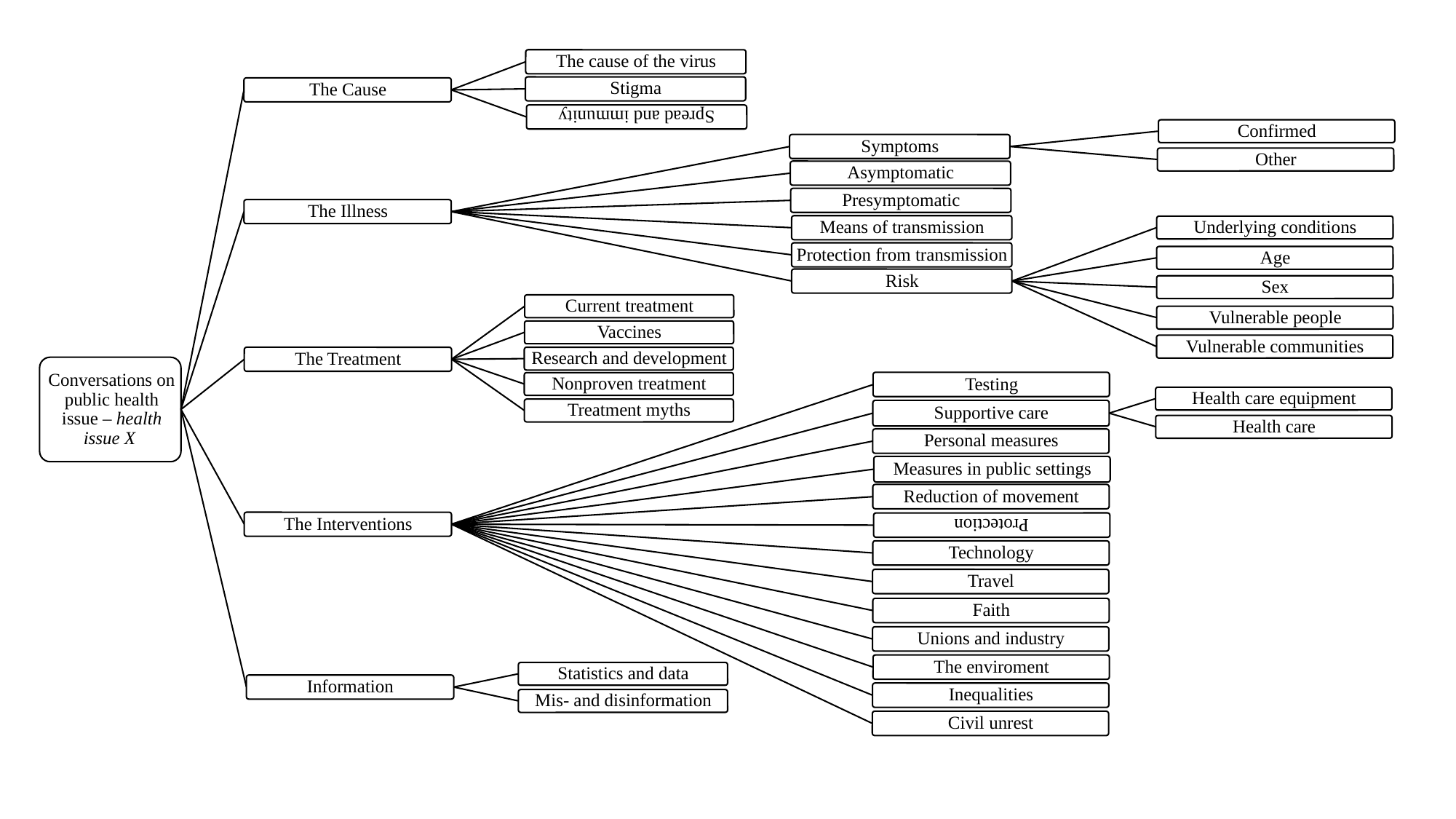

## Slide 3
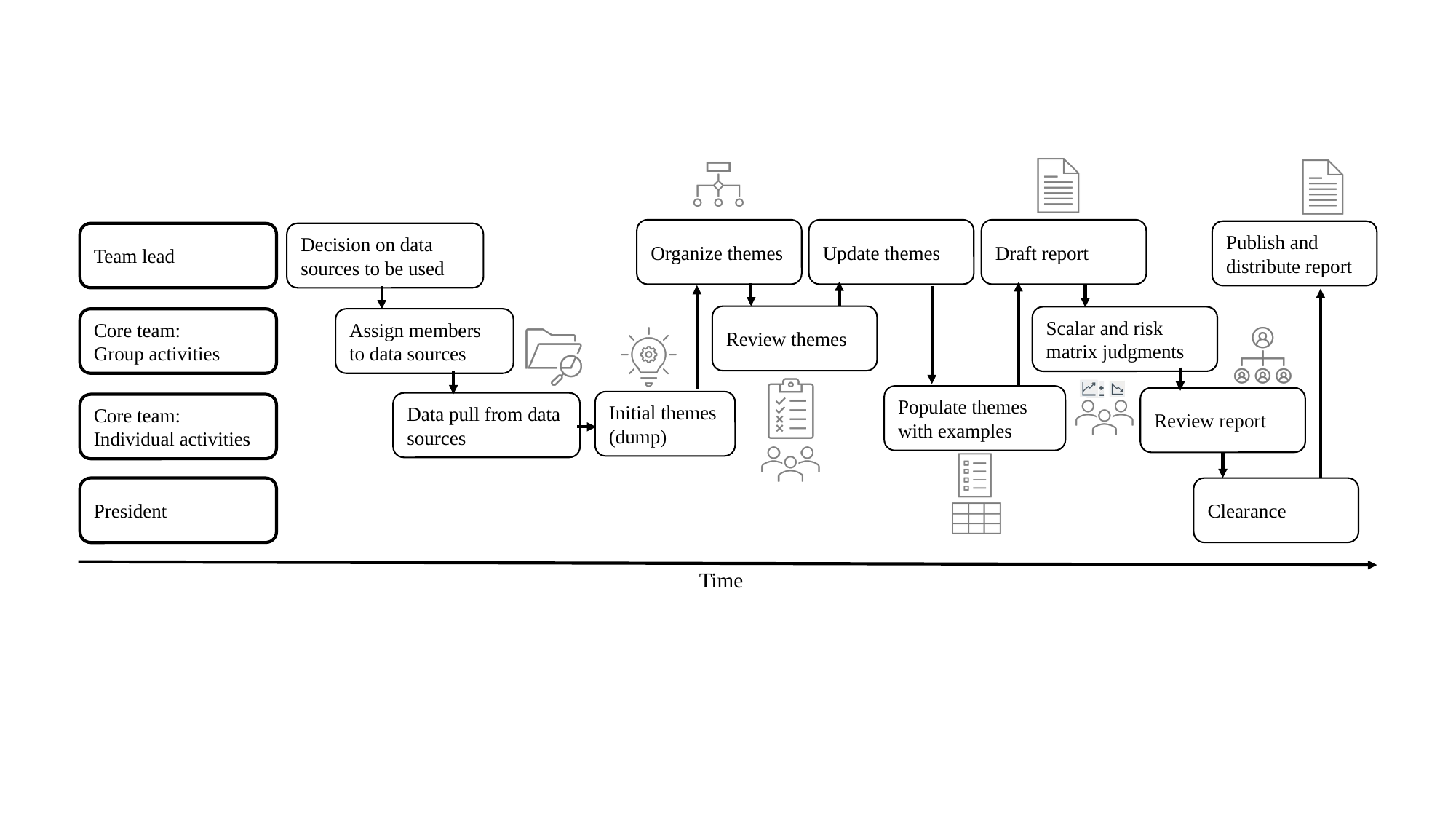

Organize themes
Update themes
Draft report
Publish and distribute report
Team lead
Decision on data sources to be used
Review themes
Scalar and risk matrix judgments
Core team:
Group activities
Assign members to data sources
Populate themes with examples
Review report
Initial themes (dump)
Data pull from data sources
Core team: Individual activities
President
Clearance
Time

## Slide 4
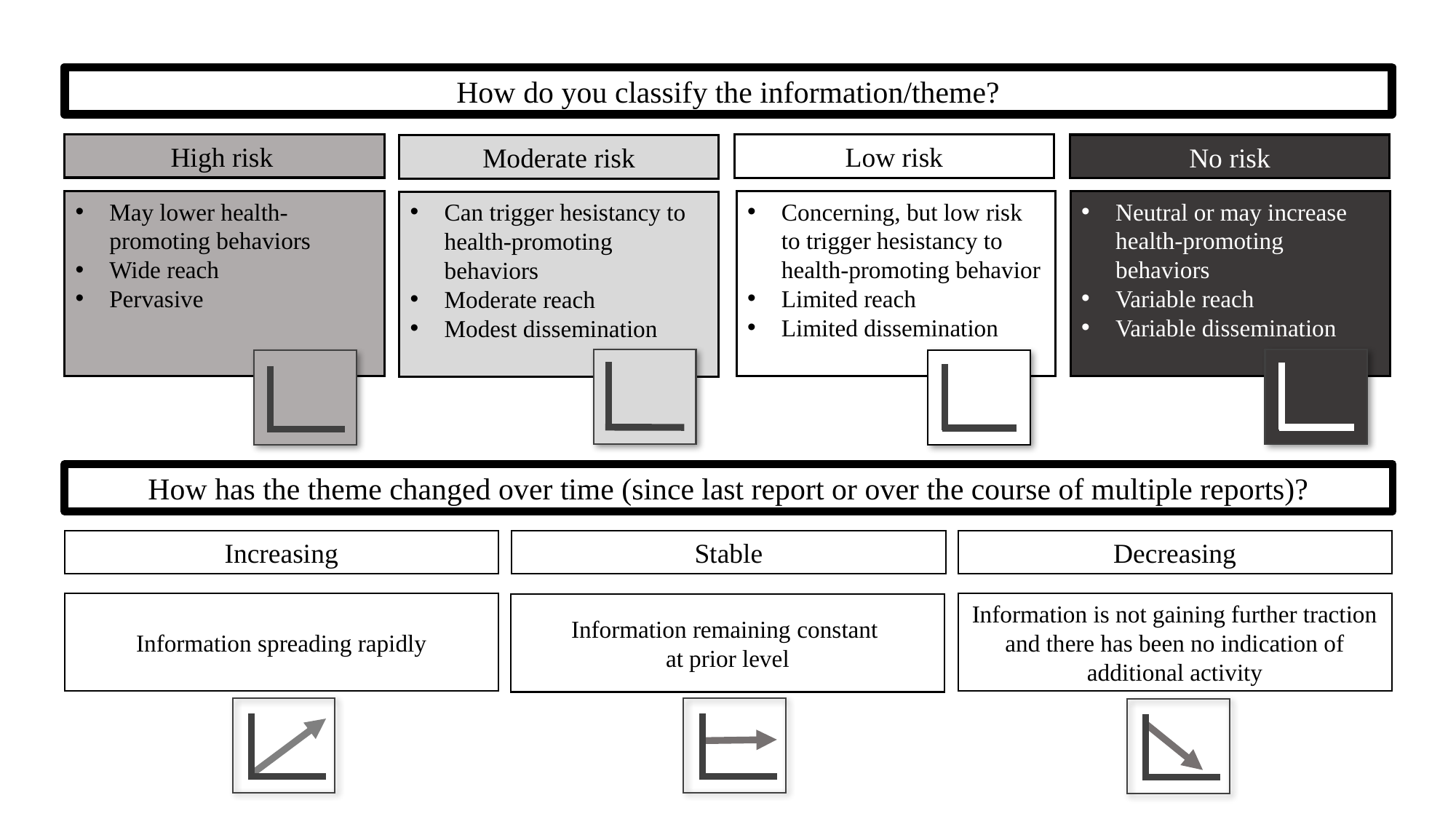

How do you classify the information/theme?
High risk
Low risk
No risk
Moderate risk
May lower health-promoting behaviors
Wide reach
Pervasive
Concerning, but low risk to trigger hesistancy to health-promoting behavior
Limited reach
Limited dissemination
Neutral or may increase health-promoting behaviors
Variable reach
Variable dissemination
Can trigger hesistancy to health-promoting behaviors
Moderate reach
Modest dissemination
How has the theme changed over time (since last report or over the course of multiple reports)?
Stable
Increasing
Decreasing
Information is not gaining further traction and there has been no indication of additional activity
Information spreading rapidly
Information remaining constant
at prior level

## Slide 5
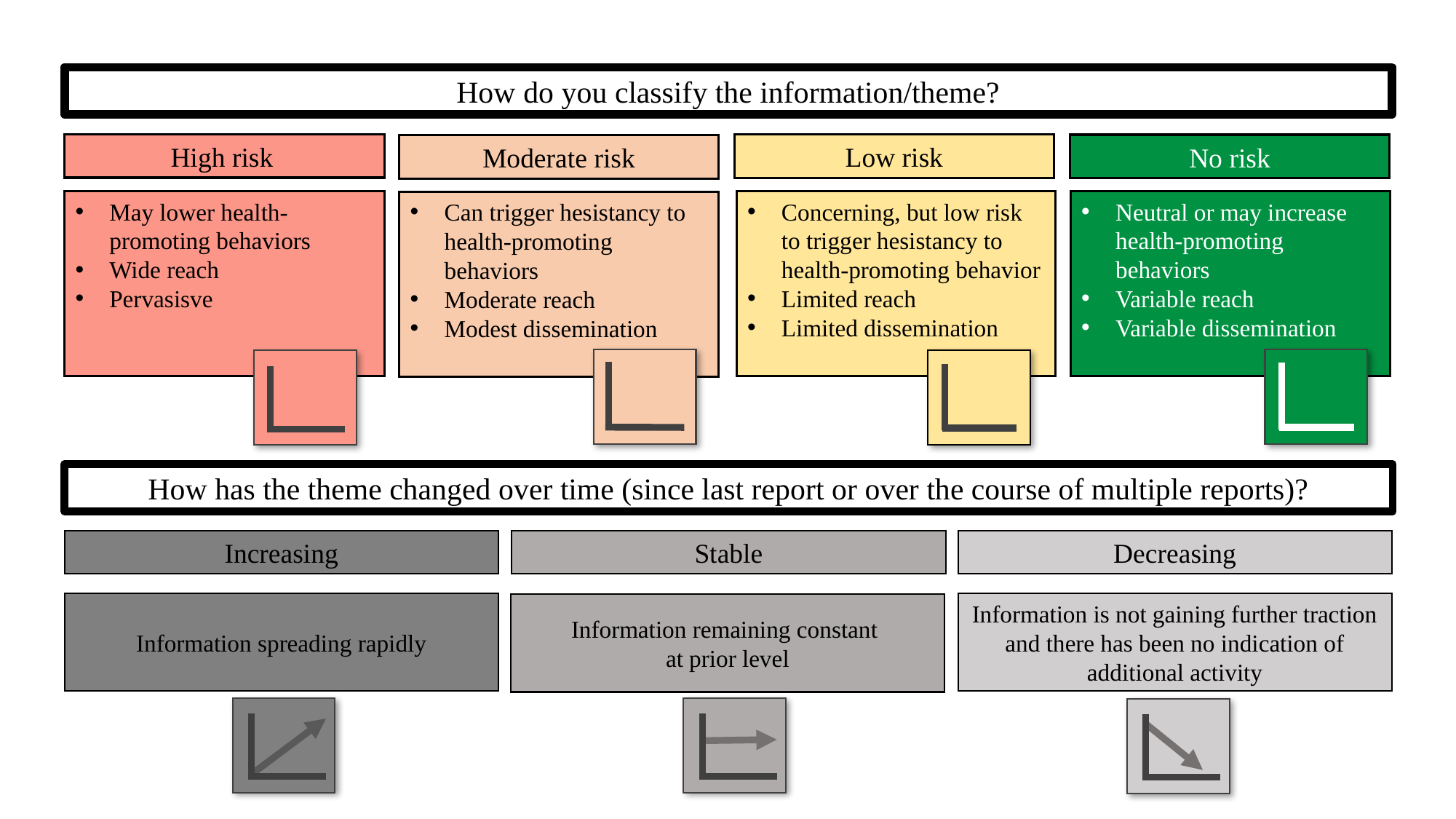

How do you classify the information/theme?
High risk
Low risk
No risk
Moderate risk
May lower health-promoting behaviors
Wide reach
Pervasisve
Concerning, but low risk to trigger hesistancy to health-promoting behavior
Limited reach
Limited dissemination
Neutral or may increase health-promoting behaviors
Variable reach
Variable dissemination
Can trigger hesistancy to health-promoting behaviors
Moderate reach
Modest dissemination
How has the theme changed over time (since last report or over the course of multiple reports)?
Stable
Increasing
Decreasing
Information is not gaining further traction and there has been no indication of additional activity
Information spreading rapidly
Information remaining constant
at prior level

## Slide 6
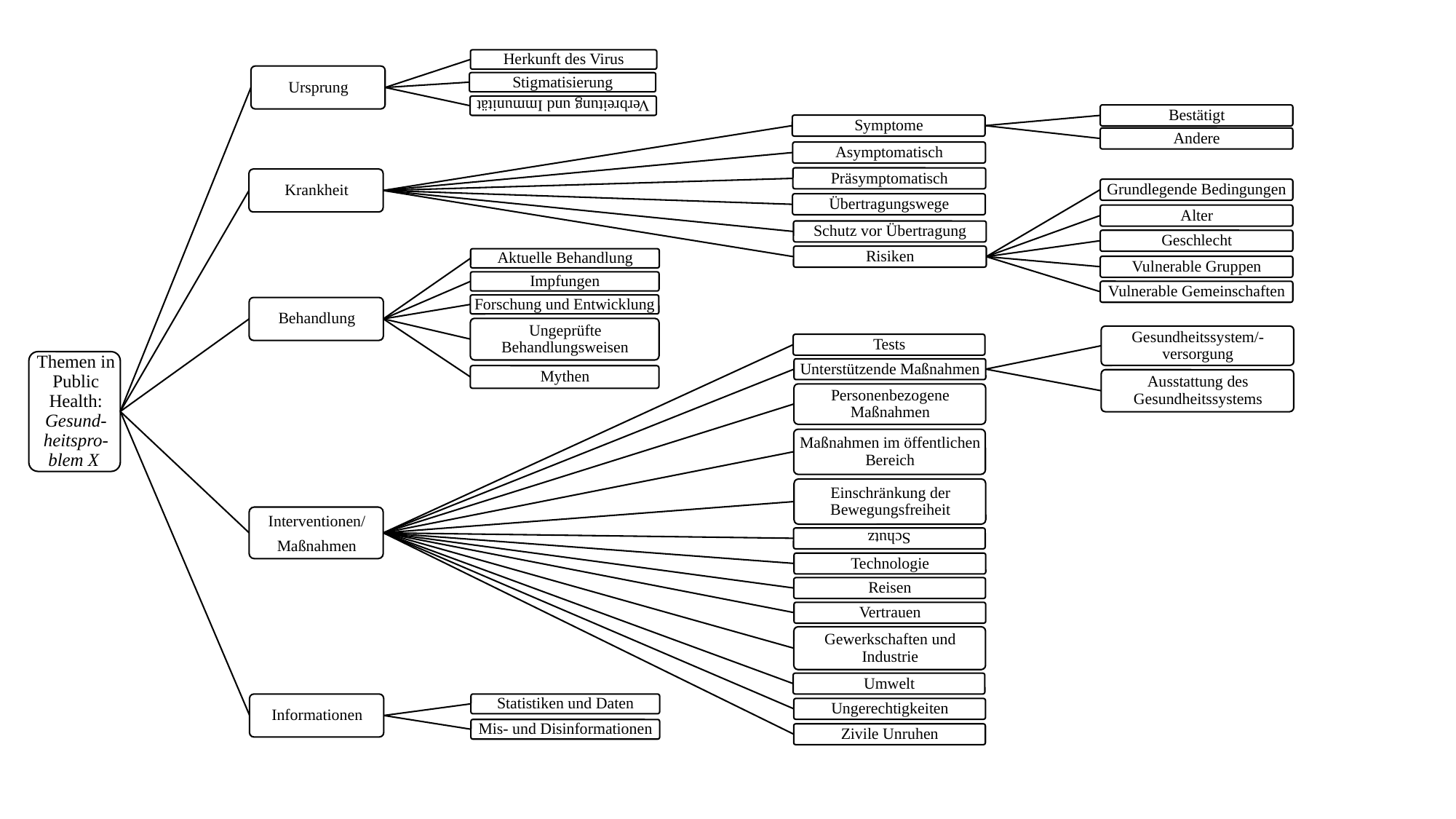

Supplement: Multimedia Appendix 4 [file infodemiology_v3i1e43646_app4.pptx]
